# Evaluating Therapeutic Effects of ADHD Medication Objectively by Movement Quantification with a Video-Based Skeleton Analysis

**DOI:** 10.3390/ijerph18179363

**Published:** 2021-09-04

**Authors:** Chen-Sen Ouyang, Yi-Hung Chiu, Ching-Tai Chiang, Rong-Ching Wu, Ying-Tong Lin, Rei-Cheng Yang, Lung-Chang Lin

**Affiliations:** 1 Department of Information Engineering, I-Shou University, Kaohsiung 840, Taiwan; ouyangcs@isu.edu.tw (C.-S.O.); asd456987321@gmail.com (Y.-H.C.); 2 Department of Computer and Communication, National Pingtung University, Pingtung 912, Taiwan; cctai@mail.nptu.edu.tw; 3 Department of Electrical Engineering, I-Shou University, Kaohsiung 840, Taiwan; rcwu@isu.edu.tw; 4 St. Dominic Catholic High School, Kaohsiung 802, Taiwan; yingtong940@gmail.com; 5 Departments of Pediatrics, Kaohsiung Medical University Hospital, Kaohsiung Medical University, Kaohsiung 807, Taiwan; rechya@kmu.edu.tw; 6 Department of Pediatrics, School of Medicine, College of Medicine, Kaohsiung Medical University, Kaohsiung 807, Taiwan

**Keywords:** attention-deficit/hyperactivity disorder, skeleton detection, OpenPose, variance, autocorrelation coefficients

## Abstract

Attention-deficit/hyperactivity disorder (ADHD) is the most common neuropsychiatric disorder in children. Several scales are available to evaluate ADHD therapeutic effects, including the Swanson, Nolan, and Pelham (SNAP) questionnaire, the Vanderbilt ADHD Diagnostic Rating Scale, and the visual analog scale. However, these scales are subjective. In the present study, we proposed an objective and automatic approach for evaluating the therapeutic effects of medication in patients with (ADHD). The approach involved using movement quantification of patients’ skeletons detected automatically with OpenPose in outpatient videos. Eleven skeleton parameter series were calculated from the detected skeleton sequence, and the corresponding 33 features were extracted using autocorrelation and variance analysis. This study enrolled 25 patients with ADHD. The outpatient videos were recorded before and after medication treatment. Statistical analysis indicated that four features corresponding to the first autocorrelation coefficients of the original series of four skeleton parameters and 11 features each corresponding to the first autocorrelation coefficients of the differenced series and the averaged variances of the original series of 11 skeleton parameters significantly decreased after the use of methylphenidate, an ADHD medication. The results revealed that the proposed approach can support physicians as an objective and automatic tool for evaluating the therapeutic effects of medication on patients with ADHD.

## 1. Introduction

Attention-deficit/hyperactivity disorder (ADHD) is a common neuropsychiatric disorder; it affects 3–5% of children worldwide [1]. The long-term impact of attentional and hyperactivity problems may lead to difficulties in several dimensions, including conduct disorders, peer relationship difficulties, educational problems and underachievement, employment problems, a lack of involvement in social activities, suicidal behaviors, and criminality [2,3,4,5,6,7,8]. If left untreated, ADHD may not only affect the patients’ functionality in childhood but may also cause social and educational impairments later in life. Therefore, early diagnosis and treatment of ADHD is crucial [9]. ADHD treatment includes drug therapy, behavioral therapy, or a combination thereof; according to neurochemical evidence, drug therapy is more effective than behavioral therapy [10]. Stimulants are widely used for ADHD drug therapy, but approximately 20% of children do not respond to them [11]. The therapeutic effects of ADHD medication can be evaluated using several methods, including the Swanson, Nolan, and Pelham (SNAP) questionnaire [12], the Vanderbilt ADHD Diagnostic Rating Scale [13], and visual analog scales [14]. Among them, the SNAP questionnaire is the most popular one and is composed of three subscales, including inattention, hyperactivity–impulsivity, and oppositional defiant disorder symptoms. However, these methods, including SNAP, are parent- or teacher-rated scales, leading to potential subjective bias. Therefore, an objective method to measure these therapeutic effects is essential for monitoring the treatment effects of ADHD.

In recent years, technology of motion detection has advanced to an extent that the device can precisely record data for long periods. For more detailed and objective motion analysis in clinical settings, marker-based systems such as a three-directional motion analysis system and accelerometer are employed. Studies have used many three-dimensional motion analysis devices, such as the VICON motion system in patients with hip osteoarthritis and in those with iliotibial band syndrome or Optotrak to detect the range of motion in healthy participants [15,16,17]. However, these marker-based systems have disadvantages such as expensive equipment and the requirement of time and technical skills for attaching sensors [18]. Increased activity is characteristic of patients with ADHD [19,20]. Murillo et al. have compared the head movements between children with ADHD and healthy controls by a computer-based infrared motion tracking system and actigraphy. They found that ADHD groups had a significantly higher number of head movements, displacement, head area, and temporal scaling than controls [21]. However, the infrared motion tracking system can only be used with sensors attached to body parts; it is a contact method. In addition, through actigraphy analysis, movement counts from the actigraphs in patients with ADHD must be converted into kilocalories (kcal) [22]; the calculation method is thus indirect. In other actigraphy studies, authors only demonstrate the classification performance between patients with ADHD and controls, or show increased mean activity in ADHD children; however, detailed movement analysis of ADHD is lacking [23,24,25]. OpenPose is a novel deep learning algorithm that has become a critical method for human posture tracking [26]. It is a real-time system for detecting body, foot, hand, and facial feature points (in total 135 feature points) on a single image without contact to subjects [26,27]. This allows the use of a simple camera to obtain patients’ images when they sit on a medical chair in the consulting room, facilitating the precise detection of patient movement before and after medication. In this study, we evaluated the therapeutic effect of ADHD medication and analyzed the movements of patients with ADHD by using the OpenPose system.

## 2. Patients and Methods

### 2.1. Participants

We included 25 children with ADHD. They were examined by a pediatric neurologist or psychiatrist and were asked to sit on a medical chair for video recording. Children with a history of epilepsy, intellectual disability, drug abuse, head injury, or psychotic disorders were excluded. ADHD diagnosis was made according to the Diagnostic and Statistical Manual of Mental Disorders (DSM)-IV criteria, and the Swanson, Nolan, and Pelham Teacher and Parent Rating Scale (SNAP-IV) was used to evaluate ADHD severity. The 26 items of SNAP-IV include the 18 ADHD symptoms (nine for inattentiveness and nine for hyperactivity/impulsiveness) and 8 oppositional defiant disorder symptoms specified in the DSM-IV. Items were rated on a 4-point scale, ranging from 0 (*not at all*) to 3 (*very much*). ADHD has three subtypes, which are diagnosed depending on the symptoms at presentation: inattentive (ADHD-I: inattentive symptoms and few or no hyperactive symptoms), hyperactive/impulsive (ADHD-H: hyperactive or impulsive symptoms and few or no inattentive symptoms), or combined (ADHD-C: both inattentive and hyperactive symptoms). Written informed consent was obtained from a family member or legal guardian of each participant. This study was approved by the Institutional Review Board of Kaohsiung Medical University Hospital (KMUIRB-SV(I)-20190060).

### 2.2. Movements Recording and Analysis

**Figure 1:**
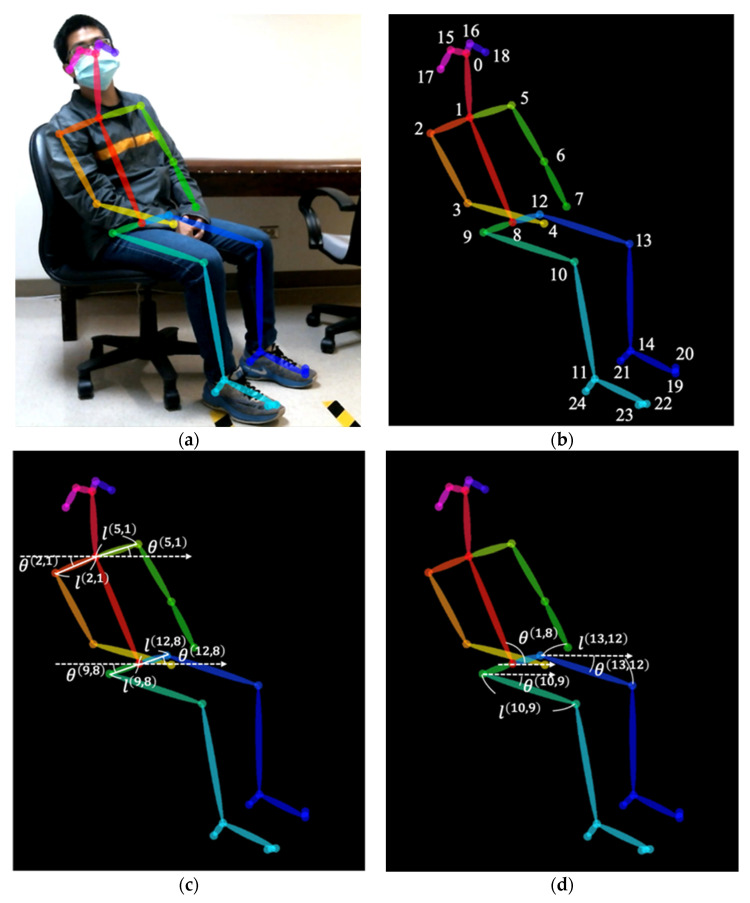
Illustration of an example of a patient’s skeleton detection. A detected patient’s skeleton represented by 25 key points and the corresponding skeleton parameters: (**a**) detected skeleton; (**b**) 25 key points; (**c**) shoulder-related and hip-related parameters; (**d**) thigh-related and trunk-related parameters.

We used a novel objective and automatic approach to evaluate the therapeutic effects of medication on patients with ADHD before and after 1 month of methylphenidate treatment, because the short-term efficacy of ADHD treatment is well documented. For example, Wilens et al. reported that teacher and parent/caregiver IOWA Conners rating scores after 1 month of methylphenidate use in children with ADHD were significantly lower than the baseline ratings for subjects who had received placebo [28]. In another study, Vance also mentioned that psychostimulants decrease ADHD symptoms, improve cognitive deficits, and decrease academic and social impairments after 4–6 weeks of use [29]. This approach was mainly based on movement quantification by autocorrelation and variance analysis of patients’ skeletons detected automatically in outpatient videos. For objective and automatic quantification, a well-known deep-learning-based library, OpenPose, was applied to detect the patient’s skeleton in each frame of the video. OpenPose is the first real-time multiperson system to jointly detect human body, hand, facial, and foot key points on single image [26]. We employed the system’s two-dimensional (2D) real-time multiperson skeleton detection functionality. Figure 1 illustrates an example of a patient’s skeleton represented by 25 key points (joints), including: the nose (0), neck (1), right shoulder (2), right elbow (3), right wrist (4), left shoulder (5), left elbow (6), left wrist (7), middle hip (8), right hip (9), right knee (10), right ankle (11), left hip (12), left knee (13), left ankle (14), right eye (15), left eye (16), right ear (17), left ear (18), left big toe (19), left small toe (20), left heel (21), right big toe (22), right small toe (23), and right heel (24). The detection result of each skeleton was represented by 2D coordinates of these 25 joints in the image space. In this study, the length of each shoulder, hip, or thigh was defined as the 2D distance between the corresponding two joints’ 2D coordinates in the image frame, instead of the physical length of shoulder, hip, or thigh. For example, the right shoulder length $l^{\left(2,1\right)}$ was calculated with the coordinates, $\left(x^{\left(1\right)},y^{\left(1\right)}\right)$ and $\left(x^{\left(2\right)},y^{\left(2\right)}\right)$, of two joints, namely, the neck (1) and right shoulder (2). Similarly, the angle of each shoulder, hip, or thigh was calculated based on the corresponding joints’ 2D coordinates in the image frame. If the ADHD child keeps a stable sitting posture during a period of time, the values of each joint’s detected coordinate (location in the 2D image frame’s coordinate domain) in the correspond series of image frames were almost the same. Therefore, the calculated values of each length-related parameter or angle-related parameter in the correspond series of image frames were almost the same, resulting a small variation of the calculated parameter values. On the contrary, if the ADHD child presents an instable sitting posture, e.g., swaying back and forth or swiveling, the values of each joint’s detected coordinate in the corresponding series of image frames were different. Therefore, the calculated values of each length-related parameter or angle-related parameter in the corresponding series of image frames were different, resulting in a large variation of the calculated parameter values. In other words, values of each detected length-related or angle-related skeleton parameters vary with the movement (a series of postures) of the ADHD child in the video.

Let $\left\{\left(x^{\left(j\right)},y^{\left(j\right)}\right)|j=0,2,\ldots ,24\right\}$ be the set of 25 joints coordinates. Four skeleton parameters—right shoulder angle $\theta^{\left(2,1\right)}$, right shoulder length $l^{\left(2,1\right)}$, left shoulder angle $\theta^{\left(5,1\right)}$, left shoulder length $l^{\left(5,1\right)}$—were calculated using the neck (1), right shoulder (2), and left shoulder (5) joints as follows:

(1) $$\theta^{\left(2,1\right)}=\left|\tan^{-1}[\frac{\left(y^{\left(2\right)}-y^{\left(1\right)}\right)}{\left(x^{\left(2\right)}-x^{\left(1\right)}\right)}]\times \frac{180}{\pi }\right|,\;l^{\left(2,1\right)}=\sqrt[{2}]{{\left(x^{\left(2\right)}-x^{\left(1\right)}\right)}^{2}+{\left(y^{\left(2\right)}-y^{\left(1\right)}\right)}^{2}}$$

(2) $$\theta^{\left(5,1\right)}=\left|\tan^{-1}[\frac{\left(y^{\left(5\right)}-y^{\left(1\right)}\right)}{\left(x^{\left(5\right)}-x^{\left(1\right)}\right)}]\times \frac{180}{\pi }\right|,\;l^{\left(5,1\right)}=\sqrt[{2}]{{\left(x^{\left(5\right)}-x^{\left(1\right)}\right)}^{2}+{\left(y^{\left(5\right)}-y^{\left(1\right)}\right)}^{2}}$$

Similarly, four skeleton parameters—right hip angle $\theta^{\left(9,8\right)}$, right hip length $l^{\left(9,8\right)}$, left hip angle $\theta^{\left(12,8\right)}$, left hip length $l^{\left(12,8\right)}$—were calculated using the middle (8), right (9), and left hip (12) joints. Two skeleton parameters—right thigh angle $\theta^{\left(10,9\right)}$ and right thigh length $l^{\left(10,9\right)}$—were calculated using the right hip (9) and right knee (10) joints. Moreover, one skeleton parameter, the trunk angle $\theta^{\left(1,8\right)}$, was calculated using the neck (1) and middle hip (8) joints as follows:

(3) $$\theta^{\left(1,8\right)}=\{\begin{array}{cc} \phi , & if\;\phi \geq 0\\ \phi +180, & if\;\phi <0 \end{array}$$

where $\phi =\tan^{-1}[\frac{\left(y^{\left(1\right)}-y^{\left(8\right)}\right)}{\left(x^{\left(1\right)}-x^{\left(8\right)}\right)}]\times \frac{180}{\pi }$.

Thus, 11 skeleton parameters’ values were obtained from the detected patient’s skeleton in each video frame (Figure 1). For each skeleton parameter in a video composed of T frames, T values constituted a time series of the skeleton parameter’s values; that is, 11 time series corresponding to 11 skeleton parameters were obtained from the patient’s skeleton in a video.

Let s=(s(1),s(2),…,s(T)) be the series of a skeleton parameter’s values. The corresponding differenced series $s^{\prime }$ was defined as follows:

(4) $$s^{\prime }=\left(s^{\prime }\left(1\right),s^{\prime }\left(2\right),\ldots ,s^{\prime }\left(T-1\right)\right)=\left(s\left(2\right)-s\left(1\right),s\left(3\right)-s\left(2\right),\ldots ,s\left(T\right)-s\left(T-1\right)\right)$$

The autocorrelation function (ACF) defines how data points in a time series are related, on average, to the preceding data points [30]. The ACF was used to measure the self-similarity of the series over different delay times and, therefore, was a function of the delay or lag τ. Let $s_{\tau }$ be the delayed series of the given series s with a lag τ. The ACF was defined as:

(5) $$\rho \left(s,s_{\tau }\right)=\frac{Cov\left(s,s_{\tau }\right)}{\sqrt{Var\left(s\right)Var\left(s_{\tau }\right)}}$$

where $Cov\left(s,s_{\tau }\right)$ is the covariance between s and $s_{\tau }$, and Var(s) and $Var\left(s_{\tau }\right)$ are the variances of s and $s_{\tau }$, respectively. The values of ρ ranged from −1 to 1. The higher the value of |ρ|, the higher the correlation between S(t) and S(t+τ). ρ=1 indicated a perfect positive correlation, ρ=0 indicated no correlation, and ρ=−1 indicated a perfect negative correlation. Moreover, $\rho \left(s,s_{1}\right)$ was the first autocorrelation coefficient when τ=1. In this study, the first autocorrelation coefficients of the series of each skeleton parameter’s values and the corresponding differenced series were calculated as follows: $acf\left(s\right)=\rho \left(s,s_{1}\right)$ and $acf^{’}\left(s^{\prime }\right)=\rho \left(s^{\prime },s_{1}^{’}\right)$. Moreover, the averaged variance of the series s was calculated with a sliding window approach:

(6) $$\bar{var}\left(s\right)=\frac{1}{I}\overset{I}{\underset{i=1}{\sum }}Var\left(s_{i}\right)$$

where $s_{i}=\left(s\left(j+1\right),s\left(j+2\right),\ldots ,s\left(j+W\right)\right),\;j=\left(i-1\right)\times W$, is the subsequence of s with a window size W and I indicates the number of subsequences. Therefore, 22 features of first autocorrelation coefficients and 11 features of averaged variance, calculated from 11 skeleton parameters, were used to characterize patients’ movements.

### 2.3. Statistical Analysis

All statistical analyses were conducted using SAS (v9.3; SAS Institute Inc., Cary, NC, USA). Data are presented as means ± standard deviation. The values of SNAP scales and each feature before and after treatment were compared using a two-tailed paired *t* test. *p* < 0.05 was considered as statistically significant.

## 3. Results

The mean age of the 25 enrolled patients (21 boys and 4 girls) was 8 years 4 months ± 2 years 6 months. All boys had ADHD-C; two girls had ADHD-C, and two girls had ADHD-I. The SNAP scores obtained from the parents and teachers before and after 1 month of methylphenidate use were 42.21 ± 12.41 vs. 37.07 ± 13.77 (*p* = 0.2783) and 37.71 ± 12.00 vs. 31.64 ± 14.75 (*p* = 0.0868), respectively. The mean durations of analyses before and after treatment were 294.19 ± 122.84 and 264.77 ± 72.39 s, respectively. The SNAP scales from the parents and teachers before and after 1 month of medication use did not differ significantly. However, hyperactivity subscales from parents and teachers and the oppositional subscale from teachers were significantly lower after 1 month (Table 1).

**Figure 2:**
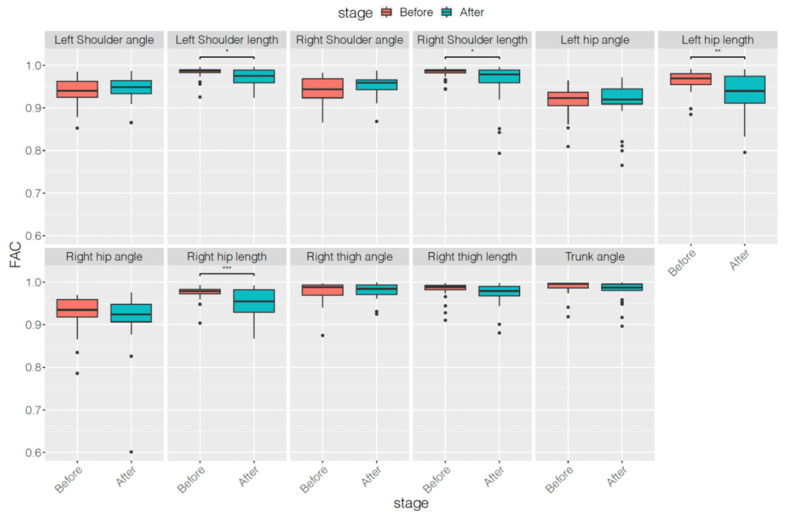
Boxplots of first autocorrelation coefficients of original series of each skeleton parameter before and after treatment. * *p* < 0.05, ** *p* < 0.01, *** *p* < 0.001.

**Figure 3:**
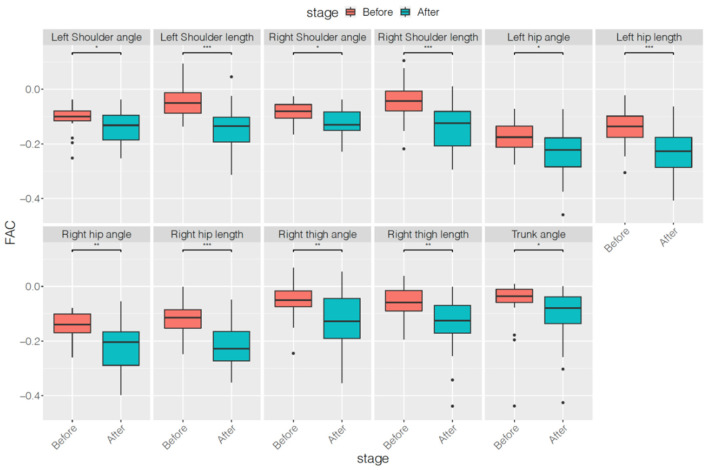
Boxplots of first autocorrelation coefficients of differenced series of each skeleton parameter before and after treatment. * *p* < 0.05, ** *p* < 0.01, *** *p* < 0.001.

**Figure 4:**
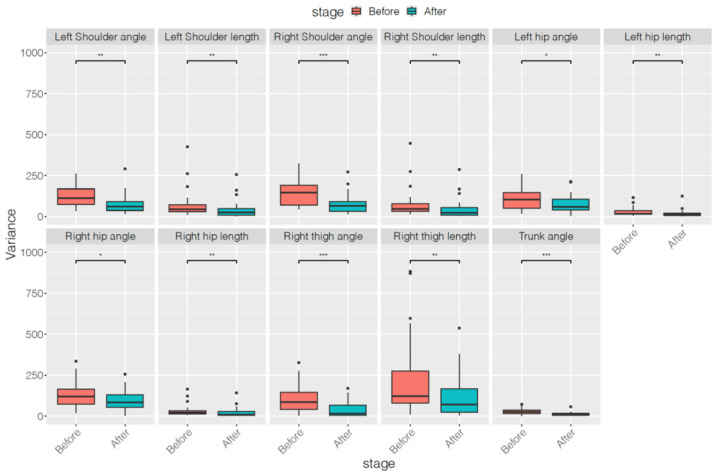
Boxplots of variances of original series of each skeleton parameter before and after treatment. * *p* < 0.05, ** *p* < 0.01, *** *p* < 0.001.

Table 2andTable 3 list the results of the first autocorrelation coefficients of the original series and differenced series, respectively, of each skeleton parameter before and after treatment. Table 4 indicates the averaged variances of the original series of each skeleton parameter before and after treatment. Only four features of the first autocorrelation coefficients of the original series—left shoulder length, right shoulder length, left hip length, and right hip length—decreased significantly after treatment. However, all 11 features of the first autocorrelation coefficients of the differenced series and all 11 features of the averaged variances of the original series decreased significantly after treatment. The features decreased most in this study were right thigh angle, length, and trunk angle of first autocorrelation coefficients of differenced series and averaged variances of original series. Moreover, when compared with the results of SNAP scales and subscales, features related to the first autocorrelation coefficients of differenced series and the averaged variances exhibited a greater reduction. To demonstrate more comparison information of statistics before and after treatment, the corresponding boxplots of the above three types of features were showed in Figure 2, Figure 3 and Figure 4.

## 4. Discussion

In this study, through the analysis of ADHD patients’ skeletons, we found four features corresponding to the first autocorrelation coefficients of the original series of four skeleton parameters; moreover, 11 features each corresponding to the first autocorrelation coefficients of the differenced series and the averaged variances of the original series of 11 skeleton parameters demonstrated a significant decrease after 1 month of methylphenidate use. Note that for each of four features corresponding to the first autocorrelation coefficients of the original series, the decrease in mean value means a decrease in the positive first-order autocorrelation of corresponding skeleton parameter’s original series (Table 2). For each of the 11 features corresponding to the first autocorrelation coefficients of the difference series, the decrease in mean value means an increase in the negative first-order autocorrelation of corresponding skeleton parameter’s difference series (Table 3). For each of the 11 features corresponding to the averaged variances of the original series, the decrease in mean value means a decrease in variation of the corresponding skeleton parameter’s original series (Table 4). Each of the aforementioned features characterized one unit of movement information of the detected patient’s skeleton, and the corresponding reduction in the feature’s value after the medication agreed with the clinical observation results of the physician. Although the total scales did not change significantly after 1 month of methylphenidate use, the hyperactivity subscales of SNAP obtained from parents and teachers and the oppositional subscale from teachers significantly decreased after 1 month. Most of our proposed features decreased more than the SNAP scales obtained from both parents and teachers did. The similarity of our proposed method and SNAP rating scale is that the quantity of movement decreased significantly after 1 month of methylphenidate use and the subscales of hyperactivity obtained from parents and teachers reduced significantly simultaneously. The main difference between software and criteria or rating scales is that our proposed method is an objective tool for evaluating therapeutic effects of ADHD. In contrast, either (DSM)-IV criteria or rating scales are subjective. They may lead to biased diagnoses or evaluations.

The SNAP questionnaire was originally developed to assess ADHD symptoms according to the DSM-III [31,32]. Although the SNAP score has high validity and reliability [33,34,35], one study found that the agreement between parent and teacher ratings is poor [12]. In addition, the parents’ ratings of inattention and hyperactivity/impulsivity are suitable predictors for research but not for clinical diagnosis. Regarding teacher ratings, only hyperactivity/impulsivity scores are suitable predictors for research and clinical diagnosis [34]. These discrepancies can lead to diagnostic uncertainty. In the present study, the hyperactivity subscales of SNAP obtained from parents and teachers and the oppositional subscale from teachers demonstrated significant decreases after 1 month of medication. We used skeleton detection to objectively evaluate the therapeutic effects of ADHD medication. Our results demonstrated that most of our proposed features had a greater decrease than that indicated by the parents’ and teachers’ hyperactivity subscales and the teachers’ oppositional subscale. Thus, our method is convenient for objectively evaluating the therapeutic effects of ADHD medications, potentially being more accurate than SNAP scores.

OpenPose is a method of localizing anatomical key points or “parts,” and it has largely been employed for identifying an individual’s body parts. This method has been used in the diagnosis and monitoring of Parkinson’s disease [36], epilepsy [37], osteoarthritis [38], and other human movements [26]. Sato et al. used OpenPose to analyze the daily clinical video recordings of patients with Parkinson’s disease recorded from the frontal angle and to convert normal gait video recordings to sequential joint coordinate data. They found a Parkinsonian gait with prominent freezing of gait and involuntary oscillations. The periodicity of each gait sequence could also be quantified by autocorrelation function-based statistical distance metrics. This metric significantly corresponded with the participants’ baseline disease statuses [36]. OpenPose was also used to track the movements in patients with epilepsy and was noted to improve posture-tracking performance in clinical environments [37]. Boswell et al. used OpenPose to replace an expensive gait analysis laboratory for detecting knee adduction moment (KAM) in patients with knee osteoarthritis. They compared KAM between 64 participants with and without osteoarthritis walking naturally and with foot progression angle modifications by 2D video analysis. Using the positions of anatomical landmarks from motion tracking, a neural network could accurately predict the peak KAM during both natural and modified walking. Their study validated the feasibility of measuring the peak KAM using positions obtainable from OpenPose analysis [38]. The most prevalent ADHD subtypes are ADHD-C and ADHD-H (78.0–81.7%), followed by ADHD-I (18.3–22.0%) [39,40,41]. In this study, 23 of 25 patients had ADHD-C; thus, most of the recruited patients demonstrated hyperactive symptoms. According to our results, the features decreased most in the present study were the right thigh angle, length, and trunk angle of first autocorrelation coefficients of differenced series and averaged variances of original series (Table 3 and Table 4). It indicates that the most frequent movements of our patients in the consulting room are swiveling and swaying back and forth. Few studies have used a noncontact method, such as Kinect, to record the amount of movement in patients with ADHD. Their results revealed significant differences in the amount of objective movement between participants with ADHD and a control group [42,43]. The present study is the first to use OpenPose to objectively analyze body movements in patients with ADHD before and after medication in the consulting room. Experimental results have shown that compared with SNAP, our proposed method may be more objective and have potential in evaluating therapeutic effects in patients with ADHD.

There is debate on the effects of methylphenidate for children with ADHD. Storebø and colleagues investigated the beneficial and harmful effects of methylphenidate as treatment for children and adolescents with ADHD [44,45]. Only small potential beneficial effects were reported when compared with methylphenidate and placebo or no intervention. The WHO Expert Committees rejected methylphenidate for ADHD application from the WHO Essential Medicines List due to uncertainties of the quality of evidence in benefit-harm viewpoints [46]. Although we do not know exactly whether or not the effects are caused by methylphenidate, in our study, the change of movement in patients with ADHD was measurable and showed significant difference before and after methylphenidate use. The relationship between our measurements and effects of medication on symptoms of ADHD patients may need to be studied further.

This study has some limitations. First, although the autocorrelation and variances of our measurements by OpenPose before and after 1 month of methylphenidate use in patients with ADHD differed significantly, the sample size was relatively small. Second, most of our cohort had ADHD-C; thus, our results may not be generalizable to all ADHD subtypes. Future studies should include a higher number of patients with different subtypes to more accurately characterize the medication effects on the three subtypes. In addition, a more accurate characterization of the different drugs in the three subtypes is also necessary. Third, children’s movements in the consulting room may be affected by uncontrollable factors, such as food intake on the day of assessment, sleep quality before the assessment, and emotion. Future studies should include a questionnaire to investigate the relationship between these confounding factors and children’s movements. Fourth, there was no placebo drug given to the patients in our study. We are not sure whether the causes of change of movement in patients are from methylphenidate or a placebo effect. In the future, an unbiased assessment of methylphenidate effect with a placebo design should be performed.

## 5. Conclusions

Most patients with ADHD have ADHD-H or ADHD-C subtype and hyperactive behaviors. In the present study, autocorrelation coefficients of the original and differenced series and averaged variances of the original series of each skeleton parameter after 1 month of methylphenidate use were used for objective evaluation of the treatment effects. OpenPose was found to be an objective and potential method for evaluating the therapeutic effects of ADHD medication, particularly in patients with ADHD-H and ADHD-C. Our proposed method appears to outperform SNAP scales in monitoring ADHD symptoms after medication use.

## Figures and Tables

**Table 1:** Comparison of subscales of SNAP in patients of ADHD before and after treatment.

Parameters	Before Treatment	After Treatment	*p* Value
Inattentiveness (P)	15.79 ± 5.36	13.71 ± 5.08	0.3307
Hyperactivity (P)	14.86 ± 5.24	11.29 ± 5.42	0.0322 *
Oppositional (P)	11.57 ± 5.33	11.36 ± 6.12	0.8944
Inattentiveness (T)	15.14 ± 4.52	15.71 ± 5.36	0.7249
Hyperactivity (T)	13.14 ± 7.64	9.21 ± 6.99	0.0476 *
Oppositional (T)	9.43 ± 5.27	6.71 ± 5.53	0.0492 *

* *p* < 0.05, P: parents, T: teacher.

**Table 2:** Comparison of first autocorrelation coefficients of original series of each skeleton parameter before and after treatment.

Parameters	Before Treatment	After Treatment	*p* Value
Left Shoulder angle	0.94 ± 0.03	0.94 ± 0.03	0.3990
Left Shoulder length	0.98 ± 0.15	0.97 ± 0.02	0.0311 *
Right Shoulder angle	0.94 ± 0.03	0.95 ± 0.03	0.4068
Right Shoulder length	0.98 ± 0.01	0.96 ± 0.05	0.0441 *
Left hip angle	0.92 ± 0.03	0.91 ± 0.05	0.6704
Left hip length	0.96 ± 0.03	0.92 ± 0.06	0.0082 **
Right hip angle	0.93 ± 0.04	0.91 ± 0.07	0.3884
Right hip length	0.97 ± 0.02	0.95 ± 0.03	0.0005 ***
Right thigh angle	0.98 ± 0.03	0.98 ± 0.02	0.7906
Right thigh length	0.98 ± 0.02	0.97 ± 0.03	0.1706
Trunk angle	0.99 ± 0.02	0.97 ± 0.03	0.1177

* *p* < 0.05, ** *p* < 0.01, *** *p* < 0.001.

**Table 3:** Comparison of first autocorrelation coefficients of differenced series of each skeleton parameter before and after treatment.

Parameters	Before Treatment	After Treatment	*p* Value
Left Shoulder angle	−0.11 ± 0.05	−0.14 ± 0.06	0.0387 *
Left Shoulder length	−0.05 ± 0.06	−0.14 ± 0.08	<0.0001 ***
Right Shoulder angle	−0.08 ± 0.04	−0.12 ± 0.06	0.0191 *
Right Shoulder length	−0.05 ± 0.07	−0.13 ± 0.09	<0.0001 ***
Left hip angle	−0.18 ± 0.06	−0.23 ± 0.09	0.0112 *
Left hip length	−0.14 ± 0.07	−0.23 ± 0.08	0.0002 ***
Right hip angle	−0.15 ± 0.05	−0.21 ± 0.10	0.0010 **
Right hip length	−0.12 ± 0.06	−0.21 ± 0.09	<0.0001 ***
Right thigh angle	−0.05 ± 0.07	−0.12 ± 0.10	0.0017 **
Right thigh length	−0.06 ± 0.06	−0.14 ± 0.10	0.0043 **
Trunk angle	−0.06 ± 0.09	−0.11 ± 0.11	0.0470 *

* *p* < 0.05, ** *p* < 0.01, *** *p* < 0.001.

**Table 4:** Comparison of averaged variances of original series of each skeleton parameter before and after treatment.

Parameters	Before Treatment	After Treatment	*p* Value
Left Shoulder angle	118.83 ± 60.41	75.38 ± 61.48	0.0042 **
Left Shoulder length	76.63 ± 92.18	43.32 ± 59.61	0.0043 **
Right Shoulder angle	141.41 ± 68.36	79.30 ± 63.18	0.0009 ***
Right Shoulder length	80.77 ± 95.89	48.93 ± 65.17	0.0072 **
Left hip angle	109.00 ± 65.89	75.56 ± 55.56	0.0220 *
Left hip length	28.70 ± 27.01	18.41 ± 25.06	0.0039 **
Right hip angle	134.14 ± 81.47	97.67 ± 65.26	0.0310 *
Right hip length	34.34 ± 38.00	22.62 ± 31.20	0.0096 **
Right thigh angle	110.61 ± 89.58	40.80 ± 47.95	<0.0001 ***
Right thigh length	295.68 ± 366.08	136.11 ± 151.64	0.0039 **
Trunk angle	28.80 ± 18.54	12.57 ± 11.64	0.0003 ***

* *p* < 0.05, ** *p* < 0.01, *** *p* < 0.001.

## Data Availability

Research data are not shared due to privacy restrictions.
